# Suicide portrayal in the Canadian media: examining newspaper coverage of the popular Netflix series ‘13 Reasons Why’

**DOI:** 10.1186/s12889-018-5987-3

**Published:** 2018-08-31

**Authors:** Victoria Carmichael, Rob Whitley

**Affiliations:** Department of Psychiatry, Douglas Mental Health University Institute, McGill University, 6875 LaSalle Blvd, Montreal, QC H4H 1R3 Canada

**Keywords:** Newspaper, Suicide, Youth, Canada, Media, Mental health, Contagion, 13 reasons why, Mixed-methods, Stigma

## Abstract

**Background:**

Evidence suggests that the media can influence societal attitudes and beliefs to various social issues. This influence is especially strong for mental health issues, particularly suicide. As such, the aim of this study is to systematically examine Canadian newspaper coverage of the popular fictional Netflix series *13 Reasons Why*, wherein the lead character dies by suicide in the final episode.

**Methods:**

Articles mentioning the series were systematically collected from best-selling Canadian newspapers in the three-month period following series release (April–June 2017). Articles were coded for adherence to key best practice recommendations on how to sensitively report suicide. Frequency counts and proportions were produced. An inductive qualitative thematic analysis was then undertaken to identify common themes within the articles.

**Results:**

A total of 71 articles met study inclusion criteria. The majority of articles did not mention the suicide method (88.7%) and did not use stigmatizing language such as ‘commit suicide’ (84.5%). Almost half of the articles linked suicide to wider social issues (43.7%) or quoted a mental health professional (45.1%). 25% included information telling others considering suicide where to get help. Our qualitative analysis indicated that articles simultaneously praised and criticized the series. It was praised for (i) promoting dialogue and discussion about youth suicide; (ii) raising awareness of youth suicide issues; (iii) shining a spotlight on wider social issues that may affect suicide. It was criticized for (i) glorifying suicide, (ii) harmfully impacting young viewers; (iii) prompting pushback from educators and schools.

**Conclusions:**

Newspaper coverage of ‘13 Reasons Why’ generally adhered to core best practice media recommendations, and sensitively discussed suicide from various angles, prompting productive discussion and dialogue about youth suicide. These findings suggest that the media can be an ally in promoting dialogue and raising awareness of important public health issues such as suicide.

**Electronic supplementary material:**

The online version of this article (10.1186/s12889-018-5987-3) contains supplementary material, which is available to authorized users.

## Background

Suicide remains a serious public health issue across the world [1], and is a leading cause of premature mortality in high-income countries such as the United States [2], Australia [3] and the United Kingdom [4]. In Canada, around 4000 people die by suicide each year, a rate of 11.5 per 100,000 [5]. This translates into roughly 70 Canadian deaths by suicide per week. Certain groups are more likely to die by suicide; the suicide rate for men is three to four times higher than for women [5], and Indigenous peoples are six to eleven times more likely to die by suicide than the general Canadian population [6].

The highest rates of suicide are seen in people aged 40 to 60. That said, young people also have high rates of suicide. In fact, suicide is the leading cause of death among people aged 10–24, and in 2009 accounted for nearly one quarter (23%) of all deaths for Canadian youth aged 15 to 19 [5]. A study examining Canadian youth suicide rates between 1980 and 2008 found a significant increase in the suicide rates for females aged 10 to 19 [7].

Given this situation, governments across the world have attempted to address the issue of suicide. The Mental Health Commission of Canada (MHCC) published Canada’s first national mental health strategy in 2012, which prioritized “promoting mental health and preventing mental illness and suicide”. The MHCC devoted resources to positive collaboration with the media as part of its strategy, aiming to improve coverage of mental health and suicide issues [8].

This action was based on considerable research indicating that the media plays a major role in shaping public attitudes, beliefs and behaviours towards health and social issues, including suicide [9]. Indeed, critical examination of media coverage of health issues has become a common form of analysis (and intervention) in public health research, with studies examining issues such as Ebola [10], Creutzfeldt-Jakob disease [11], obesity [12], diabetes [13], regulation of antibiotics [14], cancer [15], social isolation [16], mental health [17–19] and suicide [20].

With regards to suicide, some studies indicate that the media can be a positive force, educating the community and raising awareness of important suicide-related issues such as social determinants, helplines and available treatments [20]. However, other studies suggest that some media still sensationalize and simplify issues surrounding suicide, which may lead to public misunderstandings and even suicide contagion [18].

Suicide contagion is a well-documented phenomenon whereby an increase in suicide and suicidal behaviour is observed following the suicide of a celebrity, peer or family member [21]. Some research indicates that repeated sensational and glorified media coverage of a suicide can lead to an increase in contagion behaviour, particularly if it describes suicide methods in detail [22, 23]. This is especially so if the suicide involves a celebrity or public figure [24]. Indeed, a recent study found a 10% increase in suicides in the U.S. in the months following Robin William’s suicide, which was tentatively imputed to explicitly descriptive and sensational media coverage fueling ‘copycat suicides’ in the community [25].

In fact, some research shows a correlation between reporting on a suicide method and subsequent suicide by the said method [26]. Notably, evidence suggests that youth in particular are especially influenced by suicide coverage in the media, both real and fictional [27]. As such, the media can be a double-edged sword for suicide prevention. It can raise awareness and educate the public about underlying public health issues such as social determinants and available treatments; however, it may also contribute to suicide contagion.

Given this dual potentiality of the media, the MHCC recently funded the production of a short booklet containing evidence-based recommendations and best-practices for journalists reporting about mental health and suicide called *Mindset* [28]. This booklet was produced in collaboration with the Canadian Journalism Forum on Violence and Trauma and the Canadian Broadcasting Corporation, with input from senior academics familiar with the suicide prevention literature. Within *Mindset*, there are 14 recommendations to journalists for reporting suicide, such as ‘do not describe the method used’, ‘do not use the word commit’, ‘do tell others considering suicide how they can get help’ and ‘do link to wider social issues’ (see Additional file 1: Table S1).

### 13 Reasons Why

*Thirteen Reasons Why* is a popular fictional Netflix show chronicling the lives of a group of American teenagers. The lead character, Hannah Baker, experiences a number of acute and chronic stressors such as rape and bullying which lead her to take her own life in a graphic and bloody scene in the final episode. The series was released on March 31 2017 and quickly became one of the most watched series on the popular streaming platform. Indeed, it was the third most popular show on the platform across 32 countries in 2017 [29].

Beyond attracting a wide viewership, the series started a national conversation about suicide. It also prompted much media and research interest on the impact of the program and the issue of youth suicide. For instance, a just-released study found that internet searches related to suicide increased by 19% in the three-week period following the release of the series, including searches for phrases such as “how to commit suicide”, “teen suicide” and “suicide hotline numbers” [30].

Given this situation, the overall aim of this study is to systematically examine Canadian newspaper coverage of the series *13 Reasons Why*. The primary objective is to assess the content of media coverage of *13 Reasons Why* in Canadian newspapers, examining fidelity to core suicide reporting recommendations detailed in *Mindset*. The secondary objective is to identify common themes and issues discussed in these articles, especially as related to youth suicide.

## Methods

### Sampling

This study is part of a wider 8-year longitudinal study examining trends in media coverage of mental illness in Canadian media [18, 19]. The data for this study was obtained using FP Infomart. This is a comprehensive on-line depository of newspaper articles, television clips and social media posts, storing full-text news articles from over 1800 Canadian and international news sources from 1985 onwards [31]. FP Infomart is updated daily, and subscribers can search and retrieve media pieces containing key words within a specific date range. FP Infomart has been called ‘Canada’s leading media monitoring service’ [32], and is widely used in media and health research due to its ability to quickly access full-text news articles from a comprehensive list of sources [33–35].

For the present study, articles mentioning the terms ‘Thirteen/13 Reasons Why’ were collected from over 20 Canadian news sources stored in the FP Infomart database. These included Canada’s most widely-read national newspapers, such as the *Globe and Mail* and the *National Post*, as well as best-selling regional newspapers which are popular in their local communities, such as the *Toronto Star* and the *Vancouver Sun* [36]. We also included two popular online news sources: *CBC.ca News* and *metronews.*ca. A complete list of the news sources included in this study, as well as readership figures and rankings, are given in Table 1.

**Table 1:** List of newspapers and online news included in sample by circulation [36]

Rank	Title of newspaper	Average daily circulation
1.	*The Globe and Mail*	323, 133
2.	*Toronto Star*	308, 881
3.	*Maclean’s*	225,963
4.	*National Post*	183,111
5.	*Vancouver Sun*	141,246
6.	*Toronto Sun*	134,266
7.	*The Province*	124,377
8.	*Hamilton Spectator*	113,963
9.	*Calgary Herald*	113,850
10.	*Ottawa Citizen*	105,614
11.	*Winnipeg Free Press*	104,909
12.	*Edmonton Journal*	99,044
13.	*The Gazette* (Montreal)	88,654
14.	*The Record*	56,595
15.	*Winnipeg Sun*	56,211
16.	*Times Colonist*	55,152
17.	*Windsor Star*	54,119
18.	*Calgary Sun*	43,734
19.	*The Saskatoon StarPhoenix*	43,593
20.	*Edmonton Sun*	39,918
21.	*Ottawa Sun*	39,270
22.	*The Leader-Post*	36,541
23.	*The Telegram* (St. John’s)	31,823
24.	*The Daily Gleaner*	16,102
25.	*CBC.CA News* ^a^	n/a
26.	Metronews.ca^a^	n/a

*n/a* Not available

^a^These two are online news sources and so there is no daily circulation numbers available

We searched for articles in the three-month period following the series’ release on Netflix (April 1 2017 to June 30 2017). Three months was selected as a date range, as media research suggests that the 24/7 news cycle is currently very rapid, with most news articles about an event published in the days and immediate weeks following the event [37]. Indeed, other research indicates that in this era of viral news, the newsworthiness of an event decreases swiftly after the said event, to be quickly replaced by other news [38, 39]. As such, three months is a relatively lengthy time-period when analyzing news about an event in a fictional Netflix series.

### Procedures

All articles were retrieved by the first author who was closely supervised in the process by the principal investigator (the last author). Articles were excluded from analysis if the article only made a passing or incidental reference to *13 Reasons Why*. For example, articles solely about the actors from the series were excluded. This strategy allowed us to focus our attention on articles related to coverage of the series itself. Articles were also excluded if they were exact duplicates of earlier online or print versions.

The articles were then coded for the presence or absence of five key variables. These key variables were derived from core recommendations from *Mindset*, as well as questions from our wider longitudinal study. These variables were:

1. Does the article link suicide to broader social issues? (yes/no)
2. Does the article include a quote from a mental health expert? (yes/no)
3. Does the article go into details about the method of suicide used? (yes/no)
4. Does the article use the word “commit” when discussing suicide? (yes/no)
5. Does the article include information about where to get help? (yes/no)

Codes were entered into Excel for data storage and analysis. Frequency counts and proportions were produced for each variable.

We did not include every single one of the Mindset recommendations in our analysis, as many of them were not applicable, given that the suicide of Hannah Baker was fictional, not an actual suicide. For instance, “do respect the privacy and grief of family or other survivors” and “do not jump to conclusions. The reasons why people kill themselves are usually complex” are not applicable due to the fictional nature of the suicide. Similarly, other recommendations such as “do consider whether this particular death is newsworthy” and “do not shy away from writing about suicide. The more taboo, the more the myth” were not measured as they are not content recommendations, and by definition every single article would be coded positively for both of these recommendations. As such, we selected variables that were most relevant and have been linked most frequently in the scientific literature to suicide contagion and suicide prevention, as outlined in the introduction [21–26].

### Qualitative analysis

We then conducted an inductive qualitative thematic analysis of the articles. As this was an inductive analysis, no pre-existing framework was used to code and categorize the data. Instead, we followed standard procedure of identifying and labelling common themes that regularly appeared in the newspaper articles through systematic step-by-step procedures [40–42]. More specifically, both authors read all of the articles and engaged in independent ‘open-coding’, sometimes referred to as processes of ‘immersion’ and ‘mapping’ in the qualitative literature [42]. This involved labelling portions of the articles (or sometimes even whole articles) with codes that summarized key content in the articles. The authors then met to discuss their coding and mapping efforts, paying particular attention to codes that reappeared regularly across the data set in both authors’ independent analysis. At this stage, the authors consensually created a list of themes and sub-themes that best described transversal patterns in the data [41]. The first author then engaged in a second-round of supervised ‘focused coding’, utilizing the thematic framework to formally enumerate the presence or absence of the identified themes and sub-themes in each article [40, 42].

The themes and sub-themes presented in the results represent this framework, and were agreed upon consensually by both authors as a faithful summary of the common themes transversal across the data-set. This process follows recommended procedure for maintaining rigour in qualitative research through multiple coding, close teamwork and supervision, and systematic step-by-step processes of analysis [42]. Data for this study was a collection of short media articles, rather than lengthy in-depth interview or focus group transcripts. As such, the analysis was done manually rather than with computer-assisted qualitative data analysis software.

## Results

The retrieval strategy produced a grand total of 147 articles. This was reduced to 109 articles as 38 articles were excluded for making only passing or incidental reference to ‘13 Reasons Why’. Another 38 articles were subsequently excluded as duplicates during the coding process. This led to a total of 71 usable articles being included in analysis. All 71 articles are listed in Appendix. The overall proportions and percentages of key variables are presented in Table 2.

**Table 2:** Basic description of newspaper variables

Variables	Number of articles	Percent (*N* = 71)
Link to wider social issues	31	43.7
Mental health expert quoted	32	45.1
Method discussed	8	11.3
Use of “commit”	11	15.5
Tell others how they can get help	18	25.4

Frequency counts indicate that articles generally adhered to the core variables selected to assess content. For instance, out of the 71 articles, 60 (84.5%) did not use the word ‘commit’ when discussing Hannah’s suicide and 63 (88.7%) did not go into detail about the method used. Further, 45.1% of articles quoted a mental health professional while 43.7% linked Hannah’s suicide to wider social issues. However, only 18 articles (25.4%) included information about where to get help for those considering suicide.

The qualitative analysis indicated that articles tended to include praise and criticism of the series in equal measure. Sub-analysis indicated that when praising the series, articles commonly mentioned that it was useful in (i) promoting dialogue and discussion about youth suicide, (ii) raising awareness for youth suicide prevention and (iii) shining a spotlight on wider social issues that may affect suicide. In contrast, when criticizing the series, articles commonly mentioned that it (i) glorified suicide, (ii) could harmfully impact or trigger impressionable young viewers and (iii) rightfully led to pushback from educators and schools. The frequency counts and percentages of themes are presented in Table 3.

**Table 3:** Themes extracted from qualitative analysis

	Number of articles	Percent (*N* = 71)
Praise		
Promoting dialogue and discussion	53	74.6
Raising awareness for suicide prevention	39	54.9
Shining a spotlight on wider social issues	38	53.5
Critiques		
Glorification of suicide	57	80.3
Potentially harmful impact on viewers	43	60.6
Pushback from educators and schools	39	54.9

### Praise of the series

A total of 53 articles (74.6%) contained praise of the series for promoting dialogue and discussion about youth suicide. Many noted that the series created a starting point for discussion about difficult topics, particularly relating to youth mental health and suicide. For example, one article stated that “the show has prompted thousands of young people to talk about the issues of bullying, assault and suicide that they see in their own lives” (A70) and “this show is letting the genie out of the bottle. Mental health concerns are being talked about like never before” (A24). Others remarked more generally on the need for, and importance of, having an open discussion about mental health and suicide in light of the series, writing that the series has led to “hope and belief that the dialogue on and around 13 Reasons Why will create the pro-active dialogue on suicide that we, young and old alike, need so badly…” (A36). Similarly, another article mentioned that the series is “shining light on a tragic problem [mental health and suicide]” and that it is “worth talking about” (A60).

Likewise, 39 articles (54.9%) contained praise of the series for raising awareness about youth suicide issues, especially prevention. Many used the series as an opportunity to educate readers about youth suicide, presenting informative facts and figures. For example, one article informed readers that “the suicide rate among girls has increased by 38% over the past 10 years” (A60). Others also provided readers with tips for suicide prevention, placing a strong emphasis on social support, outreach and help-seeking behaviour. This included quoting mental health professionals about how to approach someone who is suicidal. For example, one quoted Mara Grunau, executive director of the Centre for Suicide Prevention, saying (A69):

‘If you’re ever worried about a friend or family member, if somebody in your world is considering suicide, ask them directly…If they answer yes, don’t panic, don’t try to solve problems, don’t be like the guidance counsellor in the Netflix series. Actively listen, affirm them, be there for them and connect them to help.’

Others provided readers with mental health resources available in their local communities (including on-line resources) or a suicide hotline number at the end of the article.

Finally, much of the media coverage used the series to shine a spotlight on various social issues related to suicide experienced by youth today. In fact, 38 articles (53.5%) highlighted the positive depiction of relevant social issues in the series, remarking that “13 Reasons speaks to young people frankly about difficult contemporary issues like cyberbullying, sexual harassment, date rape and drug use” (A24). Some articles framed this depiction of social issues as a silver lining of the series. One article, for example, wrote how “Shayna King, a 14-year-old student at Westdale Secondary School, says while the show was ‘upsetting and dark,’ it tackles realistic subject matter that could help parents and educators understand teenagers” (A16). Another noted that although the series has serious flaws, “it does show the ways schools fail to deal with issues like bullying, mental health, rape culture and sexual violence” (A40).

### Criticism of the series

A total of 57 articles (80.3%) criticized the graphic portrayal of Hannah Baker’s suicide, sometimes noting that this was gratuitously explicit. For example, one article noted that the suicide is “graphic and it is brutal…The wrist-slashing seems gratuitous and unnecessary…That scene –and the series more generally– breaks all the rules” (A24). Other articles argued that the whole storyline glorifies and romanticizes suicide. Emblematic examples included “she’s getting more quote-unquote attention than when she was alive” (A45) and “self-harm can make an unpopular girl the centre of attention” (A67). Many also critiqued the show as overly simplistic and narrow, portraying suicide as a way to “exact revenge” (A45, A67, A24). Some framed this focus on revenge as a failed opportunity to discuss mental illness and the role that it can play in suicide, writing that “another problem with the show is that it uses a suicide revenge plot instead of addressing the mental illness that caused it” (A44).

Linked to the above, 43 articles (60.6%) highlighted how the series could be damaging for vulnerable youth. The vast majority of these articles focused on the danger of contagion associated with the graphic and detailed portrayal of suicide. Indeed, a common narrative that emerged was that the show could be a potential trigger for suicidal thoughts and actions in vulnerable youth. For example, one article remarked that the series makes suicide look “quick”, “relaxing” and “even provides a how-to”, which “for teens who are actually contemplating suicide, it’s dangerous” (A44). Others described an increase in suicide threats and hospitalizations among youth which was linked to the series, referring to this as the “Thirteen Reasons Why effect” (A11). One article noted concerningly that “Calls to a Hamilton crisis line have tripled in recent months and those in the mental health field say some of that is caused by the Netflix drama 13 Reasons Why” (A45). Another described a specific case where “a 12-year-old girl who had been secretly watching the show ‘was having trouble going to school. She was having panic attacks…[she] made a suicide threat…the show had been a trigger for her’” (A68).

Finally, much of the media coverage focused on pushback from educators and schools across Canada in response to the series, with 39 articles (54.9%) of discussing this pushback. This included describing the actions taken by educators aimed at preventing discussion and use of the series in schools. Some articles featured letters from school administrators which prohibited students from talking about the show during school hours, noting that “Students at an Edmonton elementary school have been ordered to refrain from discussion about 13 Reasons Why” (A15). Others remarked how teachers have been discouraged from using the show as an educational tool or talking about it with students, writing “Ontario educators are currently being advised not to use the series in classrooms, and not to initiate conversations about the show unless first approached by students” (A08).

## Discussion

The key finding of this study is that Canadian newspapers generally adhered to core recommendations on reporting suicide when discussing *13 Reasons Why*. Journalists overwhelmingly avoided the word ‘commit’, and perhaps most importantly, very rarely went into detail about the method used by Hannah Baker. Likewise, almost half of the articles linked Hannah’s suicide to wider social issues (43.7%) and quoted a mental health expert (45.1%). These results could indicate a pattern of responsible reporting; many journalists used the series as an opportunity to raise awareness of social determinants and suicide prevention.

Nonetheless, there is room for some improvement. Just over half of the articles did not link suicide to wider social issues, nor included a quote from a mental health expert. These are important omissions, as such information could help raise awareness about social determinants and prevention strategies. Further, eight articles (11.3%) described the suicide method used in the series. Though this is a small number, it is troubling as research shows a correlation between media reporting on a suicide method and contagion suicide by such method [26].

Further, only around a quarter of articles (25.4%) included information about where to get help; such as a suicide hotline number, online resources or service listing for those considering suicide. This is a missed opportunity given the possibility of youth suicide contagion related to media portrayals [27]. It also stands in contrast to *Mindset* best practice recommendations which suggest providing information about available resources or crisis hotlines when reporting suicide, which could help those in distress to seek help.

The other key finding is that Canadian journalists had ambivalent opinions about the series, and its implications for viewers and society as a whole. This ambivalence parallels a wider societal discourse over the best approach to suicide prevention; action or silence. In other words, is it better to talk or to not talk about suicide? On the one hand, some research suggests that people prefer not to talk about suicide out of fear that it could lead to contagion and suicidality [43]. On the other hand, other research shows that awareness and discussion about suicide may actually reduce suicide ideation and encourage help-seeking behaviour [44].

Many experts square this circle by calling for greater discussion of suicide issues, while simultaneously advocating for responsible media reporting of suicide [28]. This is especially important given the pervasiveness of social media in the lives of youth, where information (and misinformation) easily spreads across networks and into users’ lives [45]. As such, sensitive and accurate reporting about suicide by the mainstream media could act as a counter-balance or corrective to (mis)information shared on social media. Indeed, there is a need for future research investigating the portrayal of suicide on social media and its impact on users.

Our analysis suggested that Canadian journalists used the series to promote dialogue and discussion about youth suicide issues*.* This is welcome news for public health advocates. Few public health issues have been solved with silence. On the contrary, positive media engagement with health issues can raise awareness, catalyze policy change and even affect individual health (and help-seeking) behaviours [46]. This is particularly the case when used in conjunction with other public health and health promotion strategies. For example, cigarette smoking and drunk driving have declined over the last decades following media engagement involving education, awareness and prevention [47, 48]. Likewise, open discussion in families, schools and workplaces is the basis of many high-profile Canadian campaigns to reduce stigma associated with mental illness such as ‘Bell Let’s Talk’ as well as the activities of the MHCC.

Articles reported that schools banned discussion of the series, and expressed serious concerns about its impact. The results suggest that more can be done to help schools and educators vis-à-vis best practice in suicide prevention, especially as a recent review indicated that commonly used programs were not effective, and sometimes even resulted in harm [49]. One approach, inspired by this study, could be the creation and distribution of evidence-based recommendations for teachers about how to talk about suicide and mental health with students. This may help reduce some of the confusion and ambivalence in Canadian schools regarding suicide prevention and mental health promotion.

There are some limitations to this study. Firstly, the study only included English-language Canadian newspapers. Newspapers in other jurisdictions or other languages may have given different results. Given the size and global influence of American media, a parallel study on U.S media may be an important next step, especially as this has recently been implicated in higher suicide rates following Robin Williams suicide [25]. Secondly, the study did not assess other forms of media such as television, radio, or social media, which may have given different results. Indeed, there is a pressing need for more research on the topic of social media and suicide, especially regarding its impact on young people. Thirdly, we collected three months of data. Although this provides a rigorous time-span of media coverage in the era of rapid news, new articles may have appeared since which were not included in our analysis. Fourthly, this paper is based on analysis of a fictional suicide, meaning that some of the 14 Mindset recommendations were not applicable. As such, the paper is inspired by Mindset, but is not a formal test of fidelity to all 14 recommendations, which would be better done through analysis of actual suicides.

## Conclusions

This study suggests that Canadian newspapers generally adhered to core best practice recommendations on how to sensitively report on suicide when discussing *13 Reasons Why*. The results also suggest that Canadian journalists tended to apply a critical lens in discussing the series and its implications for youth suicide and youth mental health. This involved praise and criticism in equal measure. These findings suggest that, taken in the round, the Canadian media is acting in a socially responsible manner consistent with best practice. This may have a positive effect on public beliefs, behaviours and attitudes regarding youth suicide and suicide prevention.

### Additional file

Additional file 1: **Table S1.** The 14 Mindset Recommendations for Reporting Suicide (DOCX 16 kb)

## Appendix

**Table 4:** List of newspaper articles included in sample

A01	Harris, B. All the right reasons; With Selena Gomez producing, series was a no-brainer for Katherine Langford. *Winnipeg Sun*. 2 April 2017.
A02	Schneller, J. Plenty of good reasons why you should watch this gutsy adaptation. *Metro News.* 2 April 2017.
A03	Ali, L. 13 Reasons Why is Netflix’s newest must-see series. *The Daily Gleaner*. 4 April 2017.
A04	Weidner, J. Suicide prevention experts worry about danger of show focused on teen’s death. *The Record*. 24 April 2017.
A05	CBC News. ‘13 Reasons Why’ criticized for messaging on teen suicide. *CBC.CA News.* 25 April 2017.
A06	CBC Radio’s Ottawa Morning. Ottawa Catholic School Board leery of new Netflix series 13 Reasons Why. *CBC.CA News*. 25 April 2017.
A07	Whitehouse, B. Parents beware: Experts worry Netflix’s ‘13 Reasons Why’ might glamorize teen suicide. *The Hamilton Spectator*. 25 April 2017.
A08	Samba, M. Downtown Mission urges public to use Netflix series 13 Reasons Why as way to discuss mental health, suicide. *Windsor Star*. 25 April 2017.
A09	May, D. Ottawa Catholic School Board warns teachers about 13 Reasons Why. *Metro News.* 25 April 2017.
A10	Laframboise, K. Talk to teenagers about 13 Reasons Why, urge mental health professionals and parents. *CBC.CA News.* 26 April 2017.
A11	Toronto Star. 13 Reasons Why called “dangerous” for teens with depression. *The Toronto Star*. 26 April 2017.
A12	Hank, M. Trouble bonding? Try TV. *Edmonton Journal*. 27 April 2017.
A13	Warren, *M. Ontario* Ministry of Education warns boards not to use Netflix’s 13 Reasons Why in classroom. *Metro News*. 27 April 2017.
A14	Markan, Z. Controversial show 13 Reasons Why resonates with psychologist’s teen clients. *CBC.CA News*. 28 April 2017.
A15	Bellefontaine, M. Principal orders Edmonton students not to talk about 13 Reasons Why at school. *CBC.CA News*. 28 April 2017.
A16	Hamilton Spectator. Public school board warns parents about Netflix hit ‘13 Reasons Why’. *The Hamilton Spectator.* 28 April 2017.
A17	Hamilton Spectator. The Spectator’s View: Reasons to talk about ‘13 Reasons Why’. *The Hamilton Spectator.* 28 April 2017.
A18	CBC News. Calgary Catholic schools nix Netflix series about teen suicide, 13 Reasons Why. *CBC.CA News*. 28 April 2017.
A19	Okeke, S. ‘Let your kid watch it,’ Quebec teen says of controversial 13 Reasons Why. *CBC.CA News*. 29 April 2017.
A20	Dimoff, A. B.C. schools warn of graphic content in Netflix series ‘13 Reasons Why’. *CBC.CA News.* 29 April 2017.
A21	Nathoo, Z. 13 Reasons Why: Is it ‘exposing the truth’ or a ‘primer’ on teen suicide? *CBC.CA News*. 29 April 2017.
A22	Li, W. Mental health experts emphasize suicide awareness in light of Netflix’s 13 Reasons Why. *Metro News.* 30 April 2017.
A23	Edwardson, L. ‘It could be a conversation starter’: Calgary school board open to discussing 13 Reasons Why with students. *Metro News.* 30 April 2017.
A24	Picard, A. Yes, 13 Reasons Why glorifies suicide. You should watch – and talk to your kids. *The Globe and Mail*. 30 April 2017.
A25	Proceviat, S. Morning Update newsletter: Purdue OKs settlement; Home Capital future uncertain; Oilers lose; Also: Ontario takes chance on basic income; lumber producers take hit. *The Globe and Mail.* 1 May 2017.
A26	Doyle, J. The fuss over 13 Reasons Why is absurd. *The Globe and Mail.* 1 May 2017.
A27	Kennedy, M. ‘13 Reasons’ sparks criticism of teen suicide depiction. *The Telegram.* 2 May 2017.
A28	Hunter, A. Suicide prevention spurs Regina students to action. *CBC.CA News*. 2 May 2017.
A29	Mahmood, D. RE: “13 Reasons Why” warning. *The Hamilton Spectator*. 2 May 2017.
A30	Ahsan, S. Netflix adds trigger warnings to 13 Reasons Why after Canadian school board bans series for ‘glamorizing’ suicide. *National Post*. 2 May 2017.
A31	O’Loughlin, E. Opinion: 13 Reasons Why and suicide prevention. *Montreal Gazette*. 2 May 2017.
A32	Lalonde, M. Schools concerned Netflix series may trigger or glorify suicide. *Montreal Gazette*. 2 May 2017.
A33	Devlin, M. 13 Reasons Why’s depiction of suicide sparks notes from capital region schools. *Times Colonist.* 2 May 2017.
A34	Masonic Temple to reopen as Concert Hall. *Toronto Star*. 3 May 2017.
A35	Ahsan, S. Netflix adds warnings to 13 Reasons Why. *Montreal Gazette.* 3 May 2017.
A36	Porter, J. Suicide prevention advocate sees opportunity in buzz around 13 Reasons Why. *CBC.CA News.* 3 May 2017.
A37	Friend, D. ‘13 Reasons Why’ highlights latest challenge for parents in a streaming world. *The Hamilton Spectator.* 3 May 2017.
A38	Kinsella, S. Don’t work 13 Reasons Why into your lesson plan, NLESD tells teachers. *CBC.CA News.* 4 May 2017.
A39	Andrews, T. Netflix’s ‘13 Reasons Why’ gets more trigger warnings. *The Hamilton Spectator.* 4 May 2017.
A40	Hill, T. I didn’t enjoy 13 Reasons Why, but the backlash is excessive. *The Globe and Mail.* 4 May 2017.
A41	Spodek, S. 13 Reasons Why made me feel uncomfortable – and that’s important. *The Globe and Mail.* 4 May 2017.
A42	Outhit, J. Suicide pranks lands Kitchener students in trouble. *The Record.* 5 May 2017.
A43	Rubinoff, J. The future is now, and it’s on…cassettes? *The Record*. 6 May 2017.
A44	Vella, I. A teen’s perspective on 13 Reasons Why. *Toronto Star*. 8 May 2017.
A45	CBC News. Hamilton crisis line sees spike in youth calls since 13 Reasons Why. *CBC.CA News*. 8 May 2017.
A46	Controversial 13 Reasons Why renewed for a second season. *The Daily Gleaner*. 2017 May 9.
A47	Come From Away wins key critics’ prize. *Toronto Star*. 9 May 2017.
A48	Netflix renews 13 Reasons Why. *Ottawa Citizen*. 9 May 2017.
A49	13 Reasons Why to return on Netflix. *Times Colonist*. 9 May 2017.
A50	Wilford, D. No Happy Ending; Fictional TV series on teen girl’s suicide is haunting. *Calgary Sun*. 9 May 2017.
A51	13 Reasons Why Renewed. *Winnipeg Sun.* 9 May 2017.
A52	13 Reasons Why will return for a second season. *National Post*. 10 May 2017.
A53	Edwardson, L. Here to help: Mental health resources available in Calgary schools. *Metro News.* 12 May 2017.
A54	Modjeski, M. Saskatoon school leaders briefed on Netflix series ‘13 Reasons Why’. *Saskatoon StarPhoenix.* 16 May 2017.
A55	Sonnylal, M. Fictional series, real-life lessons. *Montreal Gazette.* 17 May 2017.
A56	Bell, S. Cree Health Board calls for careful viewing of popular drama 13 Reasons Why. *CBC.CA News.* 18 May 2017.
A57	Hunt, B. Team hopes to shine light on ‘rampant’ mental health issues. *The Daily Gleaner.* 19 May 2017.
A58	Peipert, T. Tough topics; School district briefly pulls book over suicide theme. *Toronto Sun.* 19 May 2017.
A59	Ahsan, S. Colorado school district briefly pulls 13 Reasons Why from library shelves after seven recent student suicides. *National Post*. 19 May 2017.
A60	Shining light on tragic problem. *Toronto Sun.* 21 May 2017.
A61	Bradley, L. How 13 Reasons Why is creating valuable conversation about suicide. *Ottawa Citizen.* 22 May 2017.
A62	Kielburger, C & Kielburger, M. 13 Reasons Why sparks conversation; TV, movies can help break mental illness stigma, write Craig and Marc Kielburger. *Ottawa Citizen.* 23 May 2017.
A63	Amir, E. Series should be required viewing. *Montreal Gazette.* 25 May 2017.
A64	Fidelman, C. Crisis on campus: Universities struggle with students in distress. *Montreal Gazette.* 27 May 2017.
A65	Korenblum, M. I’m a psychiatrist. Here’s what’s wrong with 13 Reasons Why. *Toronto Star*. 29 May 2017.
A66	Direnfeld, G. ‘13 Reasons Why’ opens the door to crucial dialogue with teens. *The Hamilton Spectator.* 29 *May* 2017.
A67	Kingston, A. The real taboo in 13 Reasons Why. *Maclean’s*. 1 June 2017.
A68	Matteis, S. Increase in teen suicidal behaviour linked to 13 Reasons Why, Toronto counsellors say. *CBC.CA News.* 19 June 2017.
A69	Smith, A. Potent TV drama impacts mental health services. *Calgary Herald*. 26 June 2017.
A70	Don’t close ears to suicide issue. *Times Colonist*. 27 June 2017.
A71	Yahr, E. Netflix hits learning curve; Shows still must meet a certain standard, writes Emily Yahr. *Edmonton Journal.* 28 June 2017.

## Abbreviations

MHCC: Mental Health Commission of Canada
