# Supplementary material for: Suicide portrayal in the Canadian media: examining newspaper coverage of the popular Netflix series ‘13 Reasons Why’
Source: BMC Public Health. 2018 Aug 31;18:1086. doi: 10.1186/s12889-018-5987-3 (PMC6119255; doi:10.1186/s12889-018-5987-3)
Supplement: Supplementary file 1 — Table S1. The 14 Mindset Recommendations for Reporting Suicide (DOCX 16 kb) [file 12889_2018_5987_MOESM1_ESM.docx]

**Table S1** The 14 Mindset Recommendations for Reporting Suicide

| **Recommendation** |
| --- |

| - Do consider whether this particular death is newsworthy. |
| --- |
| - Do look for links to broader social issues.­­­­ |
| - Do respect the privacy and grief of family or other ‘survivors’. |
| - Do include reference to their suffering. |
| - Do tell others considering suicide how they can get help. |
| - Don’t shy away from writing about suicide. The more taboo, the more the myth. |
| - Don’t romanticize the act. |
| - Don’t jump to conclusions. The reasons why people kill themselves are usually complex. |
| - Don’t suggest nothing can be done because we usually never know why people kill themselves. |
| - Don’t go into details about the method used. |
| - Do use plain words. Say the person ‘died by suicide’, ‘killed herself’, or ‘took his own life.’ |
| - Don’t say the person ‘committed suicide’. It’s an outdated phrase implying illegality or moral failing. |
| - Don’t call suicide ‘successful’ or attempted suicide ‘unsuccessful.’ Death is not a matter of success. |
| - Don’t use or repeat pejorative phrases such as ‘the coward’s way out’ which reinforce myths and stigma. |
